# Tailored enrichment strategy detects low abundant small noncoding RNAs in HIV-1 infected cells

**DOI:** 10.1186/1742-4690-9-27

**Published:** 2012-03-29

**Authors:** Claudia F Althaus, Valentina Vongrad, Barbara Niederöst, Beda Joos, Francesca Di Giallonardo, Philip Rieder, Jovan Pavlovic, Alexandra Trkola, Huldrych F Günthard, Karin J Metzner, Marek Fischer

**Affiliations:** 1 Division of Infectious Diseases and Hospital Epidemiology, University Hospital Zurich, University of Zurich, Rämistrasse 100, CH-8091 Zurich, Switzerland; 2 Institute of Medical Virology, University of Zurich, Zurich, Switzerland; 3 This article is dedicated to the memory of Marek Fischer, who died in December 2010

**Keywords:** HIV-1, Small noncoding RNA, Antisense RNA, Hybridization capture

## Abstract

**Background:**

The various classes of small noncoding RNAs (sncRNAs) are important regulators of gene expression across divergent types of organisms. While a rapidly increasing number of sncRNAs has been identified over recent years, the isolation of sncRNAs of low abundance remains challenging. Virally encoded sncRNAs, particularly those of RNA viruses, can be expressed at very low levels. This is best illustrated by HIV-1 where virus encoded sncRNAs represent approximately 0.1-1.0% of all sncRNAs in HIV-1 infected cells or were found to be undetected. Thus, we applied a novel, sequence targeted enrichment strategy to capture HIV-1 derived sncRNAs in HIV-1 infected primary CD4^+ ^T-lymphocytes and macrophages that allows a greater than 100-fold enrichment of low abundant sncRNAs.

**Results:**

Eight hundred and ninety-two individual HIV-1 sncRNAs were cloned and sequenced from nine different sncRNA libraries derived from five independent experiments. These clones represent up to 90% of all sncRNA clones in the generated libraries. Two hundred and sixteen HIV-1 sncRNAs were distinguishable as unique clones. They are spread throughout the HIV-1 genome, however, forming certain clusters, and almost 10% show an antisense orientation. The length of HIV-1 sncRNAs varies between 16 and 89 nucleotides with an unexpected peak at 31 to 50 nucleotides, thus, longer than cellular microRNAs or short-interfering RNAs (siRNAs). Exemplary HIV-1 sncRNAs were also generated in cells infected with different primary HIV-1 isolates and can inhibit HIV-1 replication.

**Conclusions:**

HIV-1 infected cells generate virally encoded sncRNAs, which might play a role in the HIV-1 life cycle. Furthermore, the enormous capacity to enrich low abundance sncRNAs in a sequence specific manner highly recommends our selection strategy for any type of investigation where origin or target sequences of the sought-after sncRNAs are known.

## Background

One major posttranscriptional regulatory pathway, RNA interference (RNAi), is mediated by small noncoding RNAs (sncRNAs) [1]. Over recent years, the importance of the diverse classes of sncRNAs has been widely recognized and their impact on various biological processes demonstrated across a broad variety of organisms [2]. The most intensively studied class of sncRNAs are the 20-25 nucleotides long microRNAs (miRNAs) which play a crucial role in posttranscriptional regulation of gene expression [3].

Despite technological advances sncRNAs of low abundance have remained difficult to identify. To date, the most frequently employed method to derive sncRNAs is the generation of cDNA libraries encoding sncRNAs by, rather rate limiting, cloning and sequencing procedures [4]. While this technique allows the identification of sncRNAs of medium to high frequency with notable success, it remains less effective in defining low abundant sncRNAs. Alternate approaches have employed microarray- and PCR-based technologies to detect and quantify sncRNAs [4,5]. However, due to the short length of oligonucleotides used in microarrays and the target specificity of PCR, these procedures only lend themselves towards analyses where already known or predicted sncRNAs need to be detected. More recently, high-throughput sequencing techniques have been applied [6-8].

Discovery and screening for viral sncRNAs in infected cells faces two challenges: Firstly, sequence and length of these viral sncRNAs are yet unknown excluding approaches which depend on target specific amplification. Secondly, depending on the virus studied, virus-encoded sncRNAs may be of extremely low abundance. The first discovery of viral miRNAs was made in Epstein-Barr virus (EBV)-infected human cell lines [9] where 4.15% sncRNAs of EBV origin were identified. The specificity could be enhanced by employing subtractive hybridization which yielded libraries consisting of ~40% EBV derived sncRNAs [10]. A similar high abundance of viral sncRNAs was also observed in cells infected with other DNA viruses [11]. However, sncRNAs from RNA viruses have thus far proven less frequent, accounting commonly for < 1% of all sncRNAs in infected cells [12] (see also Note added in proof).

HIV-1 generates very low abundance [8,11,13] or undetected [14] sncRNAs. So far, only four sncRNAs with miRNA-like functionality have been identified in HIV-1 infected cells and mapped to domains in TAR [15,16], env [17], nef [18], and U3 [19]. The first published report on screening for sncRNAs in HIV-1 infected cells detected only two viral sncRNAs in 1,540 clones from HIV-1 infected HeLa T4^+ ^cells (0.13%). No functional property could be assigned to these HIV-1 sncRNAs and they were accordingly classified as degradation products by the authors [11]. Another study screened 600 sncRNA clones derived from HIV-1 infected cells for HIV-1 sncRNAs but found none which contained a viral sequence [14]. More recently, two independent surveys performed high-throughput sequencing of HIV-1 infected cell libraries and detected 0.26% [13] and 1.0% [8] HIV-1 sncRNAs in approximately 48,000 and 2.5 million screened sncRNAs, respectively.

As these studies highlight, identification of low abundant sncRNAs, such as HIV-1 encoded sncRNAs, requires either screening of a large number of sequences or an optimized selection protocol. Here we report on a novel selection and enrichment strategy for low abundant sncRNAs. Key to this approach is a highly effective enrichment by hybridization capture where hybridization probes covering the entire genome of the organism of interest - in our case HIV-1 - are included. This approach is highly successful in detecting low abundant HIV-1 sncRNAs in cDNA libraries obtained from HIV-1 infected primary human cells. The yield of HIV-1 sncRNAs increased from previously reported 0.1-1.0% to an average of 78.3% (± 7.6% (SD)) of total sncRNAs in several independent libraries.

Using this approach, we captured almost 900 HIV-1 sncRNAs, 216 of them distinguishable, in nine sncRNA libraries. HIV-1 sncRNAs are highly variable in terms of their lengths, location on the HIV-1 genome, and polarity. Tested sense/antisense hybrids of HIV-1 sncRNAs inhibit virus replication.

## Results

### Enrichment and selection of low abundant HIV-1 sncRNAs by hybridization capture

**Figure 1:**
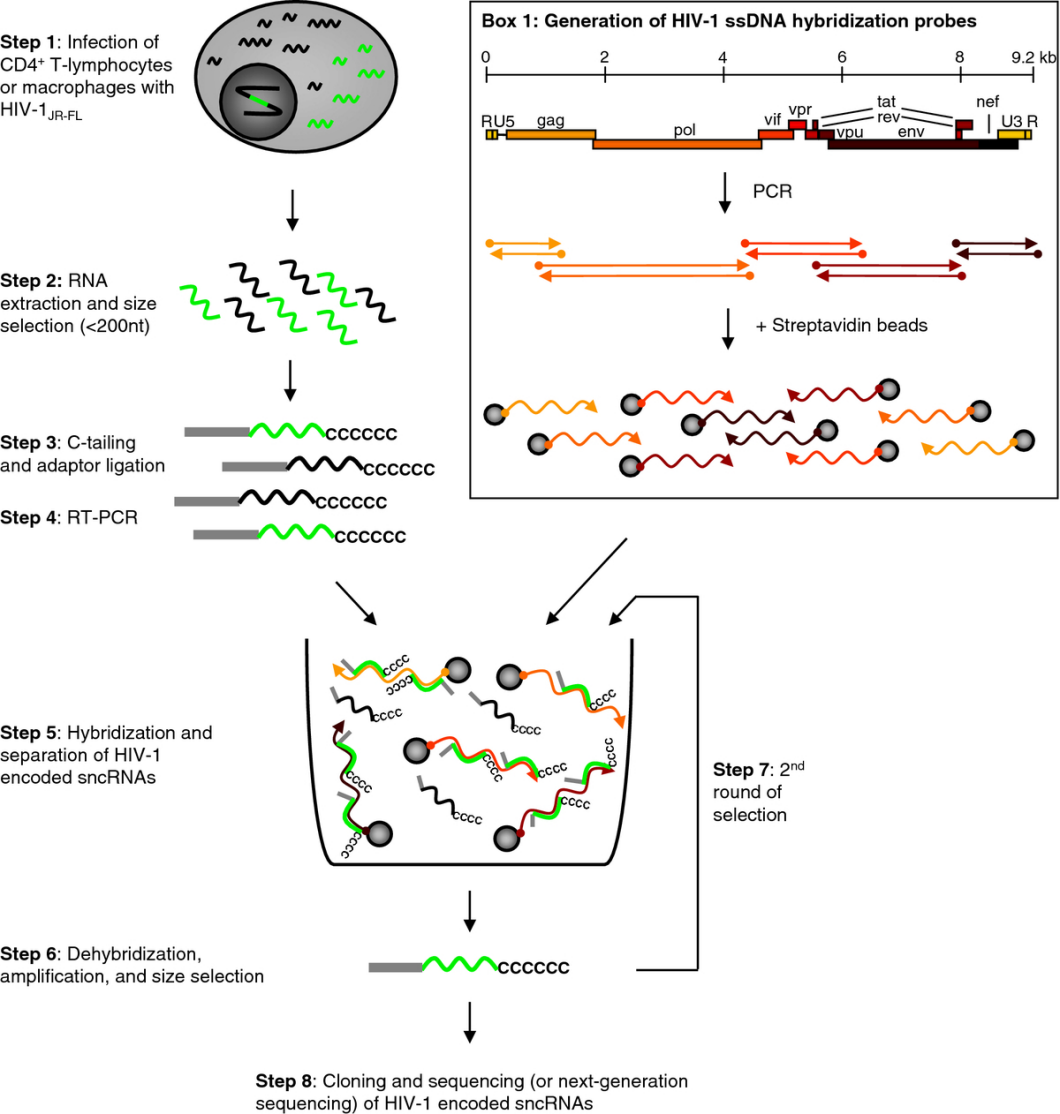
**Strategy of cDNA library generation with hybridization capture for HIV-1 encoded small noncoding RNAs (sncRNAs)**. HIV-1 susceptible cells (in our set-up, primary human macrophages or CD4^+ ^T-lymphocytes) are infected with HIV-1 (Step 1). Cellular (black) and HIV-1 encoded (bright green) sncRNAs (< 200 nt) are extracted from HIV-1 infected cells (Step 2). RNA is C-tailed at the 3'-end, adaptor-ligated at the 5'-end (Step 3), and RT-PCR is performed (Step 4). For the preparation of the HIV-1 ssDNA hybridization probes, PCR is performed with biotinylated primers for 5 overlapping regions of the genome using HIV-1_JR-FL _plasmid as template. Biotinylated amplicons are attached to streptavidin beads (Box 1). Sequences homologous to HIV-1 are enriched by incubation of cDNA derived from adaptor-ligated sncRNAs with a mixture of the 5 different HIV-1 ssDNA hybridization probes (Step 5); alternatively each HIV-1 ssDNA hybridization probe can be used separately. After hybridization capture, bound amplicons are eluted, amplified, and size selected on a gel (Step 6). The hybridization and size selection steps can be repeated (Step 7). Amplicons are cloned and sequenced or can be sequenced using next-generation sequencing technologies (Step 8).

One aim of our study was to derive an effective selection strategy for low abundant sncRNAs which would allow 1) to determine the presence or absence of sncRNAs in a given setting and 2) to allow the characterization of the full spectrum of sncRNAs generated by HIV-1 where conflicting reports have been published which suggested that either no or only extremely low numbers of HIV-1 sncRNAs are evolved in infected cells. As outlined in the following procedures, we achieved this by introducing a specific selection step which enriched for HIV-1 derived sequences. Figure 1 illustrates the various steps involved in our sncRNA selection procedure. One step is key for the success of our procedure as we enriched for HIV-1 encoded sncRNAs by specifically selecting HIV-1 sncRNAs which bound to single-stranded HIV-1 DNA in a hybridization step (Figure 1, Step 5). The HIV-1 ssDNA hybridization probes used for this purpose were generated from proviral DNA of HIV-1_JR-FL _by PCR. In total, five probes covering the entire HIV-1 genome were generated (Figure 1, Box 1). The primers used to amplify those hybridization probes were biotinylated which allowed us to couple the derived probes to streptavidin beads. Adaptor-ligated cDNA derived in Step 4 was then hybridized to the HIV-1 ssDNA hybridization probes, followed by a magnetic bead purification step to eliminate nonhybridized cDNA species (Figure 1, Step 5). The five HIV-1 ssDNA hybridization probes were either used together (as shown in Figure 1, Step 5) or in separate reactions. Both approaches proved equally effective. Bead enriched cDNA was then cloned and sequenced (Figure 1, Step 8), but could also be analyzed by next-generation sequencing technologies.

**Figure 2:**
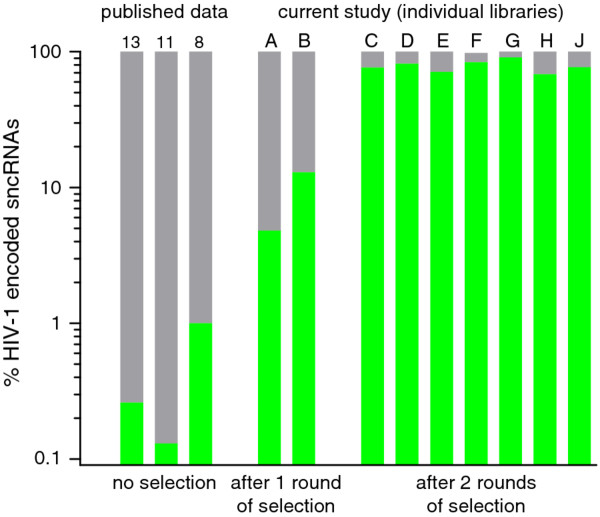
**Efficiency of hybridization capture to enrich HIV-1 encoded sncRNAs**. Comparison of published data using the currents standard protocols (left) with our novel selection strategy (right). Standard protocols with no selection led to a yield of 0.1-1.0% HIV-1 sncRNAs. Numbers above bars indicate the respective literature references [8,11,13]. Using our novel method, application of one round of hybridization capture yielded 8.9 ± 5.7% (mean ± SD) HIV-1 sncRNAs (libraries A and B). Performing two consecutive rounds of selection (libraries C-J) optimized the yield to 78.3 ± 7.6% (mean ± SD) HIV-1 sncRNAs.

We successfully employed this procedure, performing one round of selection, for two independent cDNA libraries which yielded 4.8% and 12.9% clones with sequence homology to HIV-1 (Figure 2, Additional file 1: Table S1), respectively. While the achieved enrichment for HIV-1 sncRNAs was already more than an order of magnitude higher than frequencies reported in the previously published studies, we aimed to further enrich HIV-1 sncRNAs by performing a second round of hybridization capture. We generated in total seven sncRNA libraries that underwent two consecutive hybridization selections and were all highly enriched for HIV-1 sncRNAs yielding on average 78.3% (± 7.6% (SD)) HIV-1 encoded clones (Figure 2A, Additional file 1: Table S1). These results highlight that our approach has a striking capacity to enhance the retrieval of low abundant sncRNAs. In our model system, we achieved a greater than 100-fold increase in the selection of HIV-1 encoded sncRNA species over average levels reported in the literature.

**Figure 3:**
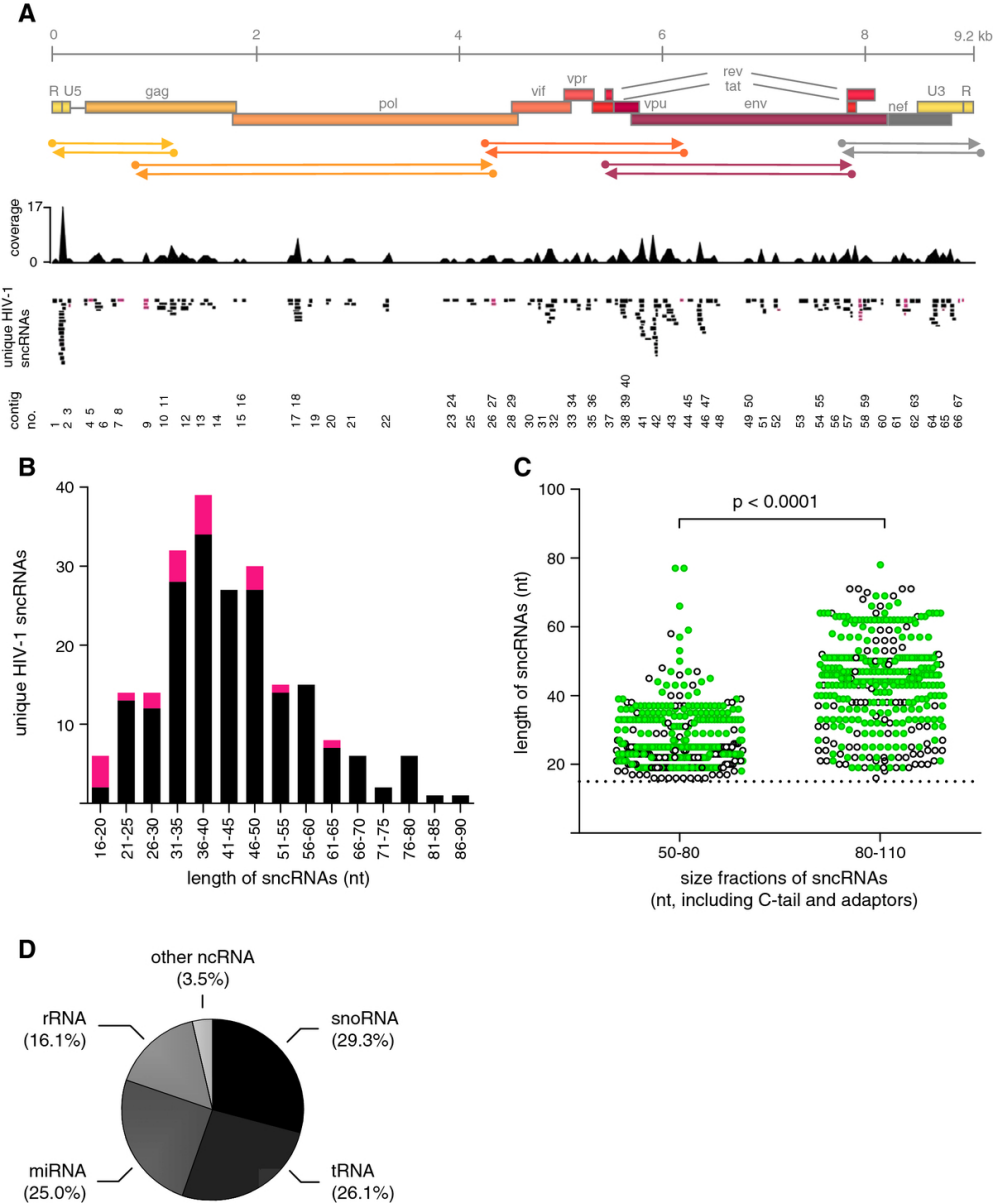
**Alignment and characterization of identified sncRNAs**. (**A**) 216 unique sncRNAs from nine libraries (Supplementary dataset 1) were aligned to the reference strain HIV-1_HXB2 _and cluster in 67 contigs distributed throughout the whole genome of HIV-1. The coverage of sncRNA per nucleotide, the single unique clones (black: sense HIV-1 sncRNAs, pink: antisense HIV-1 sncRNAs), and the contig numbers are shown. (**B**) The length (nucleotides, nt) distribution of all unique HIV-1 sncRNAs is depicted. Sense sncRNAs are shown in black, antisense sncRNAs are shown in pink. (**C**) Probing the influence of target molecule length on hybridization efficacy, libraries F, G, H, and J underwent a second size separation step before undergoing a second round of hybridization enrichment. Dehybridized cDNA was separated into two fractions of 80-110 bp or 50-80 bp length and both probed separately for hybridization efficacy. Green full circles denote HIV-1 derived sncRNAs, black open circles denote non-HIV-1 sncRNAs. Both fractions successfully retrieved sncRNA in the second round hybridization. 20-25 nucleotide long sncRNAs were retrieved from both fractions and comprised 40.1% of all sncRNAs in the small size fraction and 11.1% in the large size fraction (*p *< 0.0001, Chi square test). (**D**) Pie chart depicting the distribution of different human cellular sncRNAs in all libraries.

**Figure 4:**
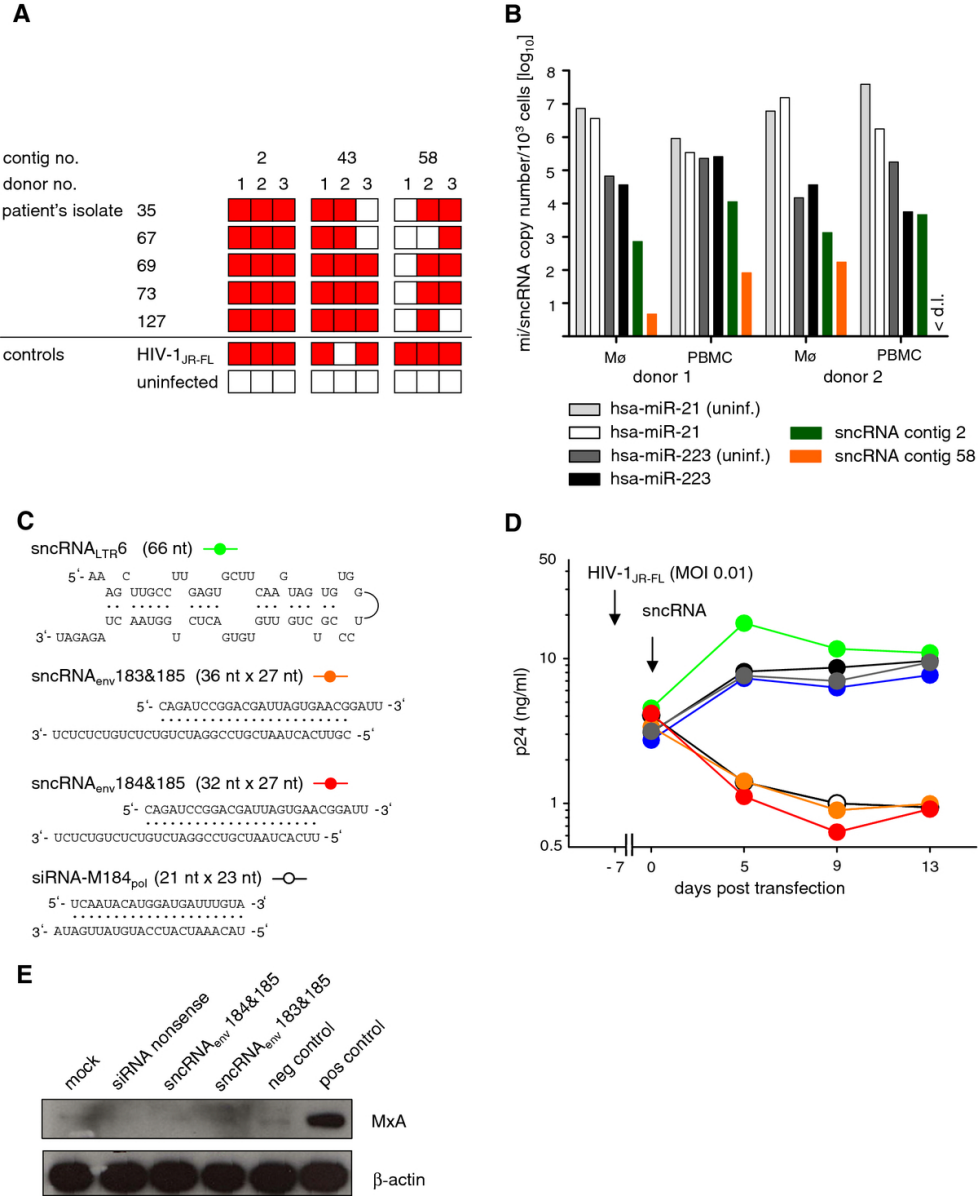
**Functional analysis of HIV-1 sncRNAs**. (**A**) CD4^+ ^T-lymphocytes of three healthy donors were infected with the indicated five different HIV-1 primary isolates and screened for the presence of HIV-1 sncRNAs in 3 contigs (contigs 2, 43, and 58) identified in HIV-1_JR-FL _infected cultures. Cell cultures from three HIV-1 negative donors were probed for each virus, and the cultures which scored positive for the respective HIV-1 sncRNAs are depicted in red squares. (**B**) Primary macrophages and CD8^+ ^T-cell depleted PBMCs of two healthy donors were infected with HIV-1_JR-FL _and HIV-1 sncRNA contig 2 (green) and contig 58 (orange) which were quantified by qPCR 14 (macrophages) and 6 (CD8^+ ^T-cell depleted PBMC) days post infection. Amplification was unsuccessful in non-infected cells (data not depicted). As controls, the cellular miRNAs hsa-miR-21 (white and light grey for non-infected cells) and hsa-miR-223 (black and dark grey for non-infected cells) were quantified. < d.l., below detection limit of 1 RNA copy/10^3 ^cells. (**C**) Predicted secondary structures of sncRNA_LTR_6, hybrids of sense and antisense orientated clones from contig 58, namely sncRNA_env_183, sncRNA_env_184 and sncRNA_env_185, and the positive control siRNA-M184 _pol _[24] (Additional file 3: Table S3) are depicted. (**D**) Inhibition of HIV-1 replication in primary macrophages by HIV-1 sncRNAs. Macrophages were infected with HIV-1_JR-FL _seven days before transfection with sncRNAs. On day 0 cells were transfected with 50 nM of the indicated sncRNA (sncRNA_LTR_6 (green), sncRNA_env_183&185 (orange), sncRNA_env_184&185 (red)) and viral replication was monitored by p24 ELISA on days 0, 5, 9, and 13 post transfection. Mock transfected (black), scrambled sncRNA (blue) and siRNA nonsense (grey) were used as negative control, and siRNA-M184 _pol _[20] (open circles) as positive control. (**E**) Western blot of primary macrophage lysates probed with anti-MxA antibody or anti-β-actin antibody as control. Primary macrophages were transfected with siRNA nonsense, sncRNA_env_183&185, and sncRNA_env_184&185 or treated with interferon-αA/D (positive control). The negative control comprised of untreated and untransfected macrophages.

To verify that the individual HIV-1 ssDNA hybridization probes selected specifically HIV-1 sncRNAs of the respective region, we generated two libraries (H and J) where HIV-1 ssDNA hybridization probes were utilized in separate reactions in the two rounds of selection. We found that 92.8 ± 7.9% (mean ± SD) of the thereby recovered HIV-1 sncRNAs were specifically enriched (Additional file 2: Table S2). Hybridization proved highly specific. Only rare false positive hybridization was observed. The latter occurred mostly amongst HIV-1 sncRNAs within the RU5 region (contig 2, Additional file 3: Table S3), the location for a highly abundant HIV-1 sncRNA contig (Figures 3A and 4B).

### Characterization of HIV-1 small noncoding RNAs

In total, we derived 1,335 clones from nine individual sncRNA libraries generated from HIV-1 infected primary cells after one or two rounds of hybridization capture (A-J, Additional file 1: Table S1). Clones were defined as valid sncRNA candidates when they 1) contained the C-tail and the 3' and 5' adaptor sequences and 2) were in the size range of > 15 and < 100 nucleotides. Eight hundred and ninety-two of these clones had a greater than 90% homology to the strain HIV-1_JR-FL _used for infection. Of these, 216 clones were distinguishable as unique clones by various measures (for example, derived from different libraries or differed in length and/or position; Additional file 3: Table S3). It can be reasoned that identical clones within one library may indicate sncRNA species which occur at higher abundance. However, deriving quantitative conclusions from our type of analysis is difficult as it cannot be ruled out that preferential amplification of certain clones occurred during PCR.

We aligned these 216 unique HIV-1 sncRNAs to the reference strain HIV-1_HXB2 _(Figure 3A, Supplementary dataset 1). They had a length of 43 ± 14 nucleotides (mean ± SD, range: 16-89 nt) (Figure 3B). Based on this alignment we found that the derived HIV-1 sncRNAs grouped within 67 different contigs, that is, single or clusters of overlapping HIV-1 sncRNAs. Forty-five contigs (67.2%) contained 2 to 17 unique sncRNAs that could represent groups of isosncRNAs similar to the recently described isomiRs [20]. Thirty-seven contigs harbored sncRNAs identified in at least two different libraries highlighting that these sncRNAs were not formed randomly. The contigs were spread throughout the HIV-1 genome, and the majority of them consisted entirely of sense sncRNAs (56 contigs, 84%). Twenty-one antisense sncRNAs were detected in either antisense only contigs (6 contigs, 9%) or in mixed sense and antisense contigs (5 contigs, 7%). Of note, sncRNAs with differential polarity in these mixed contigs have the potential to form double-stranded sncRNAs. For the 5 mixed sense/antisense contigs the double-stranded overlap ranges between 7 and 27 nucleotides (contigs no. 3, 52, 58, 62, and 65, Additional file 3: Table S3).

Due to the unexpected length of HIV-1 sncRNAs, which is longer than cellular miRNAs, we analyzed separately four libraries from two independent experiments (libraries F-J, Figure 2) where we separated the dehybridized cDNA into two fractions of 50-80 and 80-110 base pairs in length, which after subtracting the lengths of adaptors and the C-tail leads to lengths of HIV-1 sncRNAs of ≤ 25 and 25-55 bp, respectively, before subjecting the cDNA to a second round of hybridization enrichment. With this approach, we wanted to explore if the target molecule length has an influence on hybridization efficacy. The latter was a reasonable concern as it was previously suggested that short molecules are difficult to select by hybridization capture [21]. However, we could not confirm this suggestion in our setup. While as expected the separate size selection resulted in a significant difference of the median size of sncRNAs (25 nt [interquartile range, IQR: 22-33] and 44 nt [IQR: 33-51] for the 50-80 and 80-110 bp fraction, respectively, *p *< 0.0001, Wilcoxon rank sum test) (Figure 3C), the specificity of the hybridization capture for the smaller size sncRNA fraction was only slightly lower than for the larger size fraction (69.5% vs. 81.3% HIV-1 sncRNAs). One hundred forty-six of 364 (40.1%) sncRNA clones showed a length of 20-25 nucleotides in the smaller size fraction as compared to 41 of 386 (11.1%) in the larger size fraction (*p *< 0.0001, Chi square test). We can safely conclude that sncRNA clones of smaller size can also be efficiently derived using our hybridization capture. Thus, the observed length distribution of the HIV-1 sncRNAs reflects the repertoire of these small RNAs in HIV-1 infected primary macrophages and CD4^+ ^T-lymphocytes.

Our selection procedure was highly successful in both selecting a high number of HIV-1 sncRNAs and also in defining new HIV-1 sncRNA species. Of the identified 216 unique HIV-1 sncRNAs, eight correspond to previously described HIV-1 miRNAs: Six sncRNAs correspond to hiv1-miR-N367 [18] within nef, one to hiv1-miR-TAR-3p [16], and one to hiv1-miR-H1 [19]. Of particular note, while not identical in sequence and length, approximately 70% of our HIV-1 sncRNAs overlap with the 125 HIV-1 sncRNAs detected by pyrosequencing [13]. We further compared our 11 contigs consisting of antisense or sense-plus-antisense HIV-1 sncRNAs with the eight peaks within the HIV-1 antisense-derived sncRNAs very recently published by Schopman and colleagues [8]. Remarkably, three partial overlaps could be detected, all located at the 3' end (contigs 62, 65, and 67, Additional file 3: Table S3; sequences of the eight peaks were kindly provided by Nick C.T. Schopman).

Although our selection strategy for HIV-1 encoded sncRNAs is highly effective, we still retrieved approximately 30% of sncRNAs which were not of HIV-1, but mostly of human origin. Other clones contained plasmid-derived, bacterial or unknown sequences, i.e. sequences without a match in the GenBank database. The majority of those human sequences (86%) could be assigned to various classes of human cellular sncRNAs, namely, miRNAs, small nucleolar RNAs and transfer RNAs (Figure 3D). As expected, tRNA_Lys _was frequently found since this tRNA functions as primer for the initiation of reverse transcription of the HIV-1 RNA. Different cellular miRNAs have been captured; some of them show a high homology to HIV-1 and might play a role in the HIV-1 life cycle (Additional file 4: Table S4). One miRNA, hsa-miR223, has been previously described to inhibit HIV-1 replication [22].

### HIV-1 sncRNA contigs identify regions for sncRNA generation across different HIV-1 primary virus isolates

While we were successful in demonstrating that sncRNAs are generated in HIV-1 infected cells, our analysis was based on the virus isolate JR-FL. We thus sought to explore whether the identified sncRNAs are specific for this particular virus or are ubiquitously generated in HIV-1 infection. As proof-of-principle, we investigated the presence of three sncRNA contigs (contig 2, located in the conserved LTR region, and contigs 43 and 58 both located in env, Additional file 3: Table S3) in CD8^+ ^T-cell depleted PBMC from HIV-1 uninfected donors infected with five unrelated patient-derived primary virus isolates, which were obtained during acute HIV-1 infection [23]. Isolates were chosen based on the patient-specific sequences of the env gene (Philip Rieder, Beda Joos, unpublished data) to assure annealing of the specific sncRNA primers. By specifically tailored RT-PCR, all three HIV-1 sncRNA contigs were detected in most of the infected cultures (Figure 4A), indicating that these sncRNAs are not specific for an individual virus strain, nor produced randomly as they emerge upon infection with genetically divergent HIV-1 strains. Example amplicons of each contig were confirmed by sequencing (data not shown).

### Specific HIV-1 sncRNAs can inhibit virus replication

Considering the large number of HIV-1 sncRNAs we isolated and their variable length and orientation, it remains prudent to explore whether the various sncRNA species identified have functional properties. Functional screening of all 67 HIV-1 specific contigs was beyond the scope of the current analysis. Here we focused on a proof-of-principle analysis on contigs 2 and 58. These contigs were chosen based on their secondary structures which resemble precursor miRNA-like (contig 2) and siRNA-like features (contig 58). Of note, contig 58 contains sense and antisense sncRNAs. We thus chose two individual sense/antisense pairs from this contig which may form hybrids and potentially act like siRNAs (Figure 4C). The sense sncRNA is 27 nucleotides long, the antisense sncRNAs 36 and 32 nucleotides, respectively, thus, longer than cellular miRNAs or commonly used siRNAs. However, the region of double-stranded RNA is 23 and 21 base pairs long, respectively, which is within the expected range of miRNAs. To explore if these sncRNAs have any functional impact on HIV-1 replication, primary macrophages infected with HIV-1_JR-FL _were transfected with HIV-1 sncRNAs. While virus replication continued in mock, control sncRNA, and control siRNA cell cultures, HIV-1 production was potently inhibited both by the two contig 58 hybrids and the positive control siRNA-M184_pol _(Figure 4D). Both inhibited virus replication, probably using the RNA interference pathway, up to 90% compared to nonsense siRNA. To rule out that these double-stranded RNA molecules induce a non-specific interferon response, we monitored the levels of the myxovirus-resistance protein A (MxA) which is potently upregulated upon dsRNA exposure in an interferon dependent pathway [25]. None of the investigated sncRNA hybrids induced an interferon response in HIV-1_JR-FL_-infected macrophages (Figure 4E), further supporting the notion that sequence specific functions of these HIV-1 sncRNAs are responsible for the HIV-1 inhibitory activity. In contrast, the single-stranded, hairpin forming sncRNA_LTR_6 had no effect on virus replication in primary macrophages in the probed setting (Figure 4D). This preliminary analysis does not allow us to define the latter as mere degradation product because we cannot rule out functional properties of this sncRNA, for instance, during earlier steps of virus replication.

While the transfection experiments allowed us to verify the effect of the probed sncRNAs on HIV-1 infection, quantification of natural occurring sncRNA levels in unmodified cells is required to define if and at what levels these RNA molecules can be found in infected cells. To obtain a first insight on the physiological levels of HIV-1 specific sncRNAs, we quantified HIV-1 sncRNA contigs 2 (the most abundant of the HIV-1 sncRNA contigs we identified) and 58 in HIV-1_JF-RL _infected primary macrophages and CD8^+ ^T-cell depleted PBMCs from two donors. We detected HIV-1 sncRNA contig 2 in both macrophages and CD8^+ ^T-cell depleted PBMC at levels (713-4,615 RNA copies/1,000 cells; Figure 4B) comparable to low abundant cellular miRNAs [22,26]. As reference, the highly abundant cellular miRNAs hsa-miR-21 and hsa-miR-223 were quantified in parallel in these samples (Figure 4B). As expected, levels of HIV-1 sncRNA contig 58 were markedly lower than those of HIV-1 sncRNA contig 2 in both macrophages and CD8^+ ^T-cell depleted PBMC (< 1-172 RNA copies/1,000 cells). Of note, since it is likely that only a fraction of the cells are infected at the time of HIV-1 sncRNA quantification, the absolute copy number of HIV-1 sncRNAs may be higher in infected cells. Furthermore, it must be considered that the copy numbers of these contigs could potentially be underestimated, since it was not feasible to generate primers and probes similarly covering all members of the contigs. The 17 HIV-1 sncRNAs of contig 2 do not have a common overlap; thus, the chosen primer can hybridize to the majority of these HIV-1 sncRNAs, but not to all (Additional file 3: Table S3). For contig 58, the antisense but not the sense HIV-1 sncRNAs were quantified.

## Discussion

Here, we report on a novel, highly efficient selection method for sncRNAs of low abundance. Detection of low abundance sncRNAs has proven technically very challenging which may lead to an underestimation or lack of evidence for low abundant sncRNAs. HIV-1 encoded sncRNAs were detected at very low frequencies of 0.1-1.0% in previous studies [8,11,13], or were undetected [14]. Our novel strategy relies on the introduction of a crucial selection step for sncRNAs homologous to HIV-1. We achieved this by adding a hybridization capture step into an improved cloning protocol for identifying sncRNAs. The hybridization capture was performed with HIV-1 ssDNA hybridization probes, covering the whole HIV-1 genome, that were attached to streptavidin beads. Applying two rounds of hybridization capture enabled us to enrich the frequencies of selected low abundant HIV-1 sncRNAs more than 100-fold over what has been reported [8,11,13]. Importantly, more than 70% of all obtained sncRNAs were of viral origin. This is a particular advantage of our strategy. While high-throughput sequencing techniques certainly have the capacity to overcome the limitations in identifying low abundant sncRNAs, it must still be considered that more than 99% of sequenced sncRNAs retrieved by random sequencing will not be of interest and very low abundant sncRNAs might still be missed. Our approach allows for sequence specific selection with high sensitivity. This is particularly highlighted by the fact that we succeeded in detecting antisense HIV-1 sncRNAs despite the fact that HIV-1 antisense transcripts are described to be generated only at extremely low rates [27-30].

Separate sncRNA libraries derived from infected primary cells were generated, in which 216 unique HIV-1 sncRNAs with a mean length of 43 nucleotides were identified. Although, only 8% of the clones were of lengths described for genuine miRNAs or siRNAs, it has to be considered that longer HIV-1 sncRNAs still may have regulatory functions as recently reported [31,32]. For instance, it is conceivable that longer HIV-1 sncRNAs might play a role in starting the transcription process or during transcription as shown for other sncRNAs > 25 nt [33]. Another possibility is that those longer HIV-1 sncRNAs represent precursor molecules of miRNAs, although precursor miRNA-like structures were predicted only for few of them. It has been postulated that short molecules are less likely to be selected by hybridization capture [21]. However, as we show here sncRNAs of lower length can also be efficiently enriched by extracting shorter RNA molecules during size selection steps. While numerous classes of longer sncRNAs have been described in the literature, there is currently no consensus on the understanding by which processes these RNAs species are generated, and it is assumed that they have a very broad spectrum of functions [33,34]. The fact that we identified longer sncRNAs of viral origin at high frequency highlights that these RNA species warrant further investigation.

Amongst all the different types of sncRNAs identified in our screen, the capture of antisense HIV-1 sncRNAs was most surprising to us. Whether or not antisense HIV-1 RNAs are generated has been highly debated in the past, and few reports on HIV-1 antisense RNAs can be found in the literature [27-30]. It has been reasoned that the generation of antisense HIV-1 sncRNA might indeed be possible and occurs via the HIV-1 promoter in the 3'LTR [29] or via cellular promoters downstream of the integration site [35].

Many questions regarding the generation of viral sncRNAs during the HIV-1 life cycle and their function can and need to be addressed based on our initial observations and findings. Most importantly the high number of sncRNAs identified raises the possibility that HIV-1 RNA degradation products were selected. Indeed this cannot be ruled out entirely and functional analysis of all sncRNAs is certainly warranted. However, it is important to note that our procedure excludes the selection of degradation products generated by the classical pathways of RNA degradation which generate fragments lacking the 3'- and 5'-end modifications necessary for C-tailing and adaptor ligation [36]. Another argument against the possibility of enriching mainly degradation products is our observation that 86% of the cellular RNAs captured by our hybridization technique belonged to different classes of cellular sncRNAs, only 9% were classified as mRNA, thus, could potentially be degradation products. Nevertheless, even if some of the discovered HIV-1 sncRNAs are degradation products, it is not excluded that they could still play a role in the replication cycle of HIV-1.

The focus of our study was the enrichment and discovery of HIV-1 encoded, low abundant sncRNAs; however, numerous cellular miRNAs hybridizing specifically to HIV-1 were also identified using our hybridization capture that might be of importance for HIV-1 replication. One of them, hsa-miR-223, has been identified as an HIV-1 inhibitory miRNA [22]. This and other HIV-1 inhibitory miRNAs are predominantly expressed in resting CD4^+ ^T-lymphocytes [22] and have been shown to be downregulated in monocyte-derived macrophages [37]. Thus, it is not surprising that we captured hsa-miR-223 once only in our set-up that screened activated CD4^+ ^T- lymphocytes and monocyte-derived macrophages.

Using the virus strain JR-FL, we retrieved a vast number of HIV-1 sncRNAs. Of particular interest for us was to define whether these sncRNAs were specific for HIV-1_JR-FL _only or were ubiquitously generated in HIV-1 infection. As proof-of-principle we investigated this question for three contigs. Notably we found that sncRNAs of all three contigs were generated in cells infected with unrelated HIV-1 primary virus isolates, thus, confirming that the generation of these RNA species is not virus strain dependent.

Many potential functional properties of HIV-1 specific sncRNAs can be envisioned with both infection enhancing or reducing capacity. Here we report on functional assessment of sncRNA candidates from two of the 67 identified contigs. The hybridizing sense and antisense HIV-1 sncRNAs of contig 58 displayed a siRNA-like HIV-1 inhibition pattern in primary macrophages. As we demonstrate here, antisense sncRNAs appear to be generated during HIV-1 infection, and thus, might have the potential to downregulate HIV-1 production. This obviously raises a number of questions: Why would HIV-1 give raise to such negative regulatory RNAs? If they act *in vivo*, would HIV-1 not rapidly escape and induce countermeasures? Or are these negative regulators necessary for a balanced virus production (for example, ascertaining appropriate generation of structural proteins) or maybe in inducing latency? Now that our novel sncRNA isolation procedure provides the means to enrich and select these types of HIV-1 sncRNAs with high efficacy, these functional analysis can be feasible.

## Conclusions

In summary, using hybridization capture for the detection of novel sncRNAs of low abundance is a highly sensitive approach. This is particularly highlighted by our efficient enrichment of low abundant sncRNAs. More than 70% of sncRNAs we identified in our HIV-1 targeted screen were indeed derived from HIV-1 RNA demonstrating a high specificity of this enrichment by hybridization capture and showing that small RNAs are generated in HIV-1 infected primary macrophages and CD4^+ ^T-lymphocytes. HIV-1 encoded sncRNAs vary in length and in their locations on the viral genome, and they have the potential to play roles in HIV-1 replication.

## Methods

### Viruses

Primary HIV-1 isolates [23] were derived from patients' peripheral blood mononuclear cells (PBMC) by co-culturing patient CD4^+ ^T-lymphocytes with stimulated, CD8^+ ^T-cell depleted PBMC as previously described [38]. Patients were enrolled in the Zurich primary HIV infection (ZPHI) study http://clinicaltrials.gov: NCT00537966, and written informed consent was obtained from all participants. Viral replication was, for all experiments, assessed from culture supernatants by p24 ELISA (adapted from [39]). TCID_50 _of primary isolates and CD8^+ ^T-cell depleted PBMC grown HIV-1_JR-FL _virus stocks was estimated as described [40].

### Cells and infection

PBMC from healthy donors were isolated, CD8^+ ^T-cell depleted, and CD4^+ ^T-lymphocytes were stimulated and cultured as described previously [40]. Cells were infected with HIV-1_JR-FL _(MOI = 0.01), harvested 7 days post infection and lysed using QIAzol lysis reagent (Qiagen).

For the generation of macrophages, primary human monocytes were isolated from CD8^+ ^T-cell depleted PBMC using positive selection with anti-CD14-coated magnetic beads (Miltenyi Biotech). Monocytes (3 × 10^6 ^cells per T25 flask) matured to macrophages in the presence of 0.02 μg/ml human M-CSF (macrophage colony stimulating factor, PeproTech). Macrophages were maintained in RPMI-1640 supplemented with 10% FCS, 1% penicillin/streptomycin, 5% MCM (macrophage conditioned medium, sterile-filtered), 5% human serum, and 0.02 μg/ml M-CSF (the latter three ingredients were added only during the first 6-8 days). After 14 days of maturation, macrophages were infected with HIV-1_JR-FL _(MOI = 0.01). After 14 days, cells were harvested and lysed using QIAzol lysis reagent (Qiagen).

### Isolation of the low molecular weight RNA fraction

Lysed cells were homogenized with QIAshredder (Qiagen), and the extraction of small RNA (< 200 nt) was performed using miRNeasy Mini Kit (Qiagen) according to the manufacturers' instructions. RNA was eluted in 40 μl RNase-free water.

### Adaptor addition and cDNA synthesis

An aliquot (15 μl) of the low molecular weight fraction of extracted RNA was C-tailed for 15 min at 37°C using 7.5 units E.*coli *Poly(A) Polymerase (New England Biolabs) and 0.75 mM CTP (Connectorate). The synthesis of C-tails was blocked by addition of 0.5 mM Cordycepin (Sigma-Aldrich) and 2.5 units E.*coli *Poly(A) Polymerase, and incubation for 15 minutes at 37°C. At the same time, C-tailed RNA was treated with 15 U DNase (Roche). Afterwards, precipitation was performed by adding 1 volume isopropanol, 0.2 M sodium acetate, and 4 μl precipitation carrier Dr. Gentle (Takara) and centrifuged for 30 min at 16°C and 16,000 g. The pellet was washed with 80% ethanol and eluted in 20 μl H_2_O. Subsequently, the 5'-end was ligated to an 2' O-methylated RNA adaptor 5'-AUCGGAACAUCCAGACAUAACA-3' using 40 U T4 RNA ligase (New England Biolabs), 4 μM adaptor RNA, and 60 U RNaseOut (Intvitrogen) (15 min at 37°C and over night at 16°C). This was followed by precipitation as described above and elution in 10 μl H_2_O. cDNA was generated using M-MuLV Reverse Transcriptase (Finnzymes) and the 3' linker primer mf331 5'-ACCAGAGTGCGAGTAGGAAGATTGGGGGGGGG-3' partly complementary to the C-tail of the RNA. Briefly, RNA and 5 μM primer were denaturated for 5 min at 95°C followed by incubation on ice for at least 2 min. The enzyme-buffer-dNTP (400 U M-MuLV Reverse Transcriptase, 2.5 mM dNTP) mixture was added, and the reaction was incubated for 60 minutes at 37°C. Amplification of 2 μl cDNA was executed with JumpStart Taq ReadyMix (Sigma-Aldrich) for 15 cycles using 1 μM 5' adaptor primer mf311 5'-ATCGGAACATCCAGACATAACA-3' and 1.5 mM MgCl_2 _(95°C-5'; 15 × (95°C-5″; 52°C-5″; 72°C-40″)).

A second round of PCR with 25 cycles was performed using 1 μl of a 1:10 dilution of the first PCR product. Again JumpStart Taq ReadyMix (Sigma-Aldrich) supplemented with 1.5 mM MgCl_2 _and 1 μM of each 5' and 3' adaptor primers mf311 and mf315 5'-ACCAGAGTGCGAGTAGGAAGATT-3' was used (95°C-2'; 25 × (95°C-5″; 52°C-5″; 72°C-20″)). Amplicons were precipitated with isopropanol and dissolved in TENT_5/200 _(10 mM Tris-HCl, 5 mM EDTA, 0.2 M NaCl, 0.1% Triton).

### Generation of HIV-1 DNA/streptavidin beads for selection of HIV-1 sncRNAs

The HIV-1_JR-FL _plasmid [41] was used as template and amplified with HIV-1 specific biotinylated primers, using the HotStartTaq Master Mix Kit (Qiagen) supplemented with 1.5 mM MgCl_2 _(95°C 15'; 40 × (95°C 10″- 57°C 10″-72°C 4'); 72°C 7'). Five amplicons were generated using the following primers that are biotinylated at the 5'-end: 1) TAR to gag (position according to HIV-1_HXB2 _(GenBank accession number K03455): 455-1,658) with the primers mf271 5'-GGTCTCTCTGGTTAGACCAGATTTGA-3' and sk39 5'-TTTGGTCCTTGTCTTATGTCCAGAATGC-3', 2) gag to pol (1,273-4,837) with mf219 5'-AAGGCTTTCAGCCCAGAAGTAATACCCATGTT-3' and mf255 5'-ATGTCTACTATTCTTTCCCCTGCA-3', 3) pol to env (4,758-6,741) with mf254 5'-CAAATGGCAGTATTCATCCACAA-3' and mf237 5'-ATTCTTCCTGATCCCCTTCACTCTCAT-3', 4) env (5,956-8,421) with mf1 5'-CTTAGGCATCTCCTATGGCAGGAA-3' and mf2 5'-TTCCTTCGGGCCTGTCGGGTCCC-3', and 5) splice acceptor 7 (sA7) to the 3'LTR (8,317-9,700) with mf214 5'-TTTTTGCTGTACTTTCTATAGTGAATAGAGTTA-3' and cr2 5'-TGACTAAAAGGGTCTGAGGGATCTCTAGTTACCAG-3'. All primers were used in a final concentration of 1 μM. The PCR products were purified (Qiaquick, Qiagen) and eluted in 10 mM Tris-HCl (pH 8.5).

Biotinylated DNA was attached to streptavidin beads (Roche). Either 400 ng of biotinylated DNA from each PCR were used separately, or in combination (5 × 400 ng) for preparation of the beads. Briefly, 25 μg beads were washed with TENT_100 _buffer (10 mM Tris-HCl, 1 mM EDTA, 100 mM NaCl, pH 7.5, 0.1% Triton), and resuspended in 75 μl 2 × TENT_100_. Denaturated amplicons (5', 95°C) were added to the beads, and the volume was adjusted to 150 μl with H_2_O. DNA was immobilized by 30 minutes incubation with the beads at 37°C. Streptavidin-biotinylated, single-stranded DNA complexes were achieved by heating to 90°C for 1 minute. The attachement-dehybridization procedure was repeated once. Streptavidin-biotinylated-ssDNA complexes were washed 3 times with TENT_1000 _(10 mM Tris-HCl, 1 mM EDTA, 1 M NaCl, pH 7.5, 0.1% Triton) and 3 times with TENT_100_. They were stored in TENT_100 _at 4°C.

### Selection of HIV-1 sncRNAs

For the hybridization of amplified HIV-1 sncRNAs to the Streptavidin-biotinylated-ssDNA complexes, 10 μl of these beads (> 10^10 ^molecules of each HIV-1 ssDNA hybridization probe) were added to the amplified HIV-1 sncRNAs and incubated for 3 minutes at 95°C followed by a cool down to 50°C over night on a head to tail wheel. Beads were washed 4 times with pre-warmed (50°C) TENT_5/200 _buffer. Annealed amplified HIV-1 sncRNAs were eluted from the beads by adding 15 μl Tris-HCl buffer (10 mM Tris-HCl, pH 8.5) and heating for 5 minutes at 95°C. Beads and eluted sncRNA were separated by magnetic separation. HIV-1 sncRNAs were amplified using JumpStart Taq ReadyMix (Sigma) supplemented with 1.5 mM MgCl_2 _and 1 μM of each adaptor-specific primers mf311 and mf315 (95°C-2'; 30 × (95°C-5″; 52°C-5″; 72°C-30″)). Amplicons were size-selected using a 3% MetaPhor agarose gel. DNA with a length of 50-110 bp was extracted from gel using GenElute Agarose Spin Columns (Sigma). When two selection steps were performed, eluate was precipitated with isopropanol and the hybridization and size selection steps were repeated. Eluates were precipitated with isopropanol and eluted in 15 ul H_2_O.

### Cloning and sequencing of HIV-1 sncRNAs

Amplified and selected HIV-1 sncRNAs were ligated into the vector pDrive using the QIAGEN PCR Cloning kit (Qiagen). Single clones were sequenced in one direction with the primer T7 using BigDye chain terminator chemistry and the automated sequencer ABI 3100 (Applied Biosystems).

Sequences were controlled for the presence of both adaptor sequences, which were subsequently deleted to obtain the sncRNA sequence. This analysis was performed using the software BioEdit [42]. All sncRNA sequences were aligned to the reference strains HIV-1_HXB2 _and HIV-1_JR-FL _using the software DNAstar (DNA-star Madison). Sequences with > 90% homology to the reference strain HIV-1_JR-FL _were considered HIV-1 specific. FASTA [43] was chosen for further nucleotide similarity searches. Classification of small RNA sequences was based on sequence analysis using the GenBank database http://www.ncbi.nlm.nih.gov/genbank/, the miRNA registry database http://www.mirbase.org/, and the human tRNA database http://gtrnadb.ucsc.edu/. Secondary structures of selected HIV-1 sncRNA were predicted with RNAstructure 5.2 [44]. SncRNA sequences smaller than 16 nucleotides were not included in our analysis.

### Statistical analyses

Statistical analyses were performed using GraphPad Prism5.0 software. The two-tailed Chi square test and the Wilcoxon rank sum test were used for binary and cardinal data, respectively. *p *< 0.05 was considered statistically significant.

### Transfection of primary macrophages with HIV-1 sncRNAs

Maturated macrophages were generated and infected with HIV-1_JR-FL _as described above. Seven days after infection cells were transfected with HIV-1 sncRNAs using jetPRIME transfection reagent (Polyplus-Transfection). Briefly, medium was replaced by Opti-MEM^® ^I Reduced Serum Media (Invitrogen) and the transfection mix was added to the cells according to the manufacturer's instructions. After four hours, 10% FCS (Invitrogen) was added. The next day the transfection medium was replaced by RPMI-1640 supplemented with 10% FCS and 1% penicillin/streptomycin. The following oligonucleotides were used for sncRNA transfection: sncRNA_LTR_6; sncRNA_env_183; sncRNA_env_184; sncRNA_env_185 (Supplementary dataset 1). Control siRNA labelled with AlexaFluor488 (AllStars negative controls, Qiagen), here named as nonsense siRNA, was used as control for the transfection efficiency and negative control for virus inhibition, whereas siRNA-M184_pol _was chosen as positive control as previously described [24]. Western blot analysis for detection of the interferon type I inducible MxA protein was carried out as previously described using a mouse monoclonal antibody directed against MxA [45].

### Detection of HIV-1 sncRNAs in cells infected with primary HIV-1 isolates

HIV-1 sncRNAs detected in most or all of the libraries were screened in terms of their presence in primary cells infected with primary HIV-1 isolates. Infection of CD8^+ ^T-cell depleted PBMC, RNA isolation, C-tailing, and reverse transcription were performed as described above. HIV-1 sncRNAs were amplified, using 1 uM of primer mf315, 1 uM of respective HIV-1 sncRNA-specific primer (mf382 5'- ATAAAGCTTGCCTTGAGTG-3', mf178 5'- ATGGTAGAACAGATGCATGAGGATATAAT-3', CA-179 5'- CGTTCACTAATCGTCCGGATCTGTC-3') and JumpStart Taq DNA Polymerase (Sigma). PCR was performed as follows: 95°C-2'; 50 × (94°C-10″; 55°C-10″; 72°C-40″). Amplicons were loaded on 3% MetaPhor agarose gel and separated by electrophoresis mobility.

### Quantification of HIV-1 sncRNAs in HIV-1 infected cells

In order to quantify mi/sncRNA in macrophages and CD8^+ ^T-cell depleted PBMC, the small RNA fraction was extracted from cell lysate, C-tailed, reverse transcripted, and amplified as described above. Mi/sncRNA were amplified by using 1 μM of the adaptor primer mf315 and 1 μM of the corresponding mi/sncRNA specific primer (hsa-miR-21 5'-TAGCTTATCAGACTGATGTTGA-3', hsa-miR-223 5'-TGTCAGTTTGTCAAATACCC-3', mf382 5'-ATAAAGCTTGCCTTGAGTG-3', VV153 5'-CGTTCACTAATCGTCCGGAT-3'). For quantification via qPCR, EvaGreen™ Dye (Biotium) was used for cellular miRNAs and 0.3 and 0.2 μM of LNA probes CA20tq 5'-tgTgTgcCcGt-3' and VV154 5'-TGTctctgtctCT-3', respectively, were used for HIV-1 derived sncRNA, LNA bases are indicated as lower case letters.

## Competing interests

The authors declare that they have no competing interests.

## Authors' contributions

CFA carried out the analysis of the sequence data, performed the alignments and the statistical analyses and wrote the manuscript. VV established the PCR for the sncRNAs, performed the transfection and HIV-1 inhibition experiments. BN prepared the libraries, cloned and sequenced sncRNAs, and performed the HIV-1 sncRNA-specific PCRs. BJ assisted in the evaluation of the sequences. FG assisted in the analysis and illustration of the data. PR provided the full length sequences of env of the primary virus isolates. JP assisted in sncRNA transfection/IFN induction experiments and provided reagents to determine interferon induction. AT participated in the design of the study and writing of the manuscript. HFG participated in the design of the study and in the analysis. KJM participated in the analysis, design of the study and writing of the manuscript. MF conceived the study, participated in its design and coordination and in the analysis. This article is dedicated to the memory of Marek Fischer, who died in December 2010. All other authors contributed to the writing of the manuscript, read and approved the manuscript.

## Note added in proof

After the submission of this paper, two publications have appeared which show that RNA viruses can efficiently and physiologically express miRNAs. Work by Kincaid *et al. *reported that bovine leukemia virus (BLV) abundantly expresses a physiological cluster of viral miRNAs [46]. A separate report by Klase *et al. *showed that HIV-1 can express a cellular miRNA inserted into its viral genome without loss of replication competence [47].

## Supplementary Material

Additional file 1 **Table S1**. Frequencies of all sncRNA clones containing the C-tail and both 3' and 5' adaptors in each library. Given are absolute numbers of HIV-1 sncRNAs, their percentages, and total of sequenced sncRNA clones. Click here for file

Additional file 2 **Table S2**. SncRNA libraries generated with separate HIV-1 ssDNA hybridization probes. Click here for file

Additional file 3 **Table S3**. Characteristics of unique HIV-1 sncRNAs. Click here for file

Additional file 4 **Table S4**. Captured cellular miRNAs. Click here for file
