# Supplementary material for: Tailored enrichment strategy detects low abundant small noncoding RNAs in HIV-1 infected cells
Source: Retrovirology. 2012 Mar 29;9:27. doi: 10.1186/1742-4690-9-27 (PMC3341194; doi:10.1186/1742-4690-9-27)
Supplement: Additional file 1 — Table S1. Frequencies of all sncRNA clones containing the C-tail and both 3' and 5' adaptors in each library. Given are absolute numbers of HIV-1 sncRNAs, their percentages, and total of sequenced sncRNA clones. [file 1742-4690-9-27-S1.PDF]

Table S1: Frequencies of all sncRNA clones containing the C-tail and both 3' and 5' adaptors in each library. Given are absolute numbers of HIV-1 sncRNAs, their percentages, and total of sequenced sncRNA clones.

			1st round of selection		2nd round of selection	
			Five HIV-1 ssDNA hybridization probes			
**experiment**	**cell source**	**HIV-1$_{JR-FL}$**	**mixed**	**separate**	**mixed**	**separate**
# 1	macrophages	+	Library A<br>13/101<br>12.9%	-	Library C<br>62/81<br>76.5%	-
# 2	CD4+ T-lymphocytes	+	Library B<br>4/84<br>4.8%	-	Library D<br>185/227<br>81.5%	-
# 3	macrophages	+	not cloned	-	Library E<br>119/168<br>70.8%	-
# 4	CD4+ T-lymphocytes	+	not cloned	not cloned	Library F<br>25/30<br>83.3%	Library H[b]<br>125/183<br>68.3%
# 5	macrophages	+	not cloned	not cloned	Library G<br>39/43<br>90.7%	Library J[c]<br>320/418<br>76.6%
# 6 [a]	macrophages	-	Library K<br>1/204<br>0.5%	-	-	-
# 7 [a]	CD4+ T-lymphocytes	-	Library L<br>0/145<br>0.0%	-	-	-
# 8 [a]	macrophages	-	not cloned	-	Library M<br>1/185<br>0.5%	-
# 9 [a]	CD4+ T-lymphocytes	-	not cloned	-	Library N<br>1/28<br>3.6%	-
# 10 [a]	macrophages	-	not cloned	-	Library O<br>8/46<br>17.4%	-

[a] for the following pairs of experiments, cells from the same donor were used: #1 and #6, #2 and #7, #3 and #8, #4 and #9, #5 and #10

[b] the following HIV-1 ssDNA hybridization probes were used separately: gag/pol (2), pol/env (3), sA7-LTR (5)

[c] all 5 HIV-1 ssDNA hybridization probes were used separately
