# Supplementary material for: Tailored enrichment strategy detects low abundant small noncoding RNAs in HIV-1 infected cells
Source: Retrovirology. 2012 Mar 29;9:27. doi: 10.1186/1742-4690-9-27 (PMC3341194; doi:10.1186/1742-4690-9-27)
Supplement: Additional file 2 — Table S2. SncRNA libraries generated with separate HIV-1 ssDNA hybridization probes. [file 1742-4690-9-27-S2.PDF]

Table S2: SncRNA libraries generated with separate HIV-1 ssDNA hybridization probes.

		HIV-1 ssDNA hybridization probes				
		1	2	3	4	5
Library		TAR-gag	gag/pol	pol-env	env	sA7-LTR
H	HIV-1 sncRNAs / total sncRNA clones (%)	n.p.	29/56 (51.8%)	83/96 (86.5%)	n.p.	13/31 (41.9%)
	HIV-1 sncRNAs corresponding to the specific HIV-1 ssDNA hybridization probe	n.p.	29 (100%)	76 (91.6%)	n.p.	10 (76.9%)
	Matching HIV-1 ssDNA hybridization probe to not corresponding HIV-1 sncRNAs			1[(a)], 4, 5		1[(a)]
J	HIV-1 sncRNAs / total sncRNA clones (%)	45/67 (67.2%)	78/86 (90.7%)	67/91 (73.6%)	63/87 (72.4%)	67/87 (77.0%)
	HIV-1 sncRNAs corresponding to the specific HIV-1 ssDNA hybridization probe	43 (95.6%)	77 (98.7%)	66 (98.5%)	54 (85.7%)	64 (95.5%)
	Matching HIV-1 ssDNA hybridization probe to not corresponding HIV-1 sncRNAs	4 or 5	1[(a)]	4 or 5	1[(a)], 5	1[(a)]

n.p. = not performed

[(a)] HIV-1 sncRNAs from the RU5 genomic region were preferentially enriched as not corresponding HIV-1 sncRNAs
