# Supplementary material for: Tailored enrichment strategy detects low abundant small noncoding RNAs in HIV-1 infected cells
Source: Retrovirology. 2012 Mar 29;9:27. doi: 10.1186/1742-4690-9-27 (PMC3341194; doi:10.1186/1742-4690-9-27)
Supplement: Additional file 3 — Table S3. Characteristics of unique HIV-1 sncRNAs. [file 1742-4690-9-27-S3.PDF]

Table S3: Characteristics of unique HIV-1 sncRNAs.

Seq No.	Library [a]	Sequence (5' - 3')	Length	Position HXB2 [b]		Polarity	Contig No.	sncRNA # of identical clones
				Start	End			
**1**	C	ATCTGAGCCTGGGAGCTCTCTGGCTAACTAGGGAACCCACTG	42	475	516	sense	1	3
**2**	H	AAAGCTTGCCTTGAGTGCTTCAAGTAGTGTGTG	33	530	562	sense	2	1
**3**	J	AAAGCTTGCCTTGTGTGCTTCAAGTAGTGTGTGCCCGTCTGTTGTGTGA	49	530	578	sense	2	2
**4**	J	AAAGCTTGCCTTGAGTGCTTCAAGTAGTGTGTGCCCGTCTGTTGTGTGACT	51	530	580	sense	2	3
**5**	J	AAAGCTTGCCTTGAGTGCTTCAAGTAGTGTGTGCCCGTCTGTTGTGTGACTCT	53	530	582	sense	2	1
**6**	J	AAAGCTTGCCTTGAGTGCTTCAAGTAGTGTGTGCCCGTCTGTTGTGTGACTCTGGTAACTAGAGAT	66	530	595	sense	2	2
**7**	J	AAGCTTGCCTTGAGTGCTTCAAGTAGTGTGTGCCCGTCTGTTGTGTGACT	50	531	580	sense	2	6
**8**	D	AGCTTGCCTTGAGTGCTTCAAGTAGTGTGTGCCCGTCTGTTGTGTGA	47	532	578	sense	2	32
**9**	B	GCTTGCCTTGAGTGCTTCAAGTAGTGTGTGCCCGTCTGTTGTGTGACTCTGGTA	54	533	586	sense	2	1
**10**	A	CTTGCCTTGAGTGCTTCAAGTAGTGTGTGCCCGTCTGTTGTGTG	44	534	577	sense	2	4
**11**	D	TGCCTTGAGTGCTTCAAGTAGTGTGTGCTCGTCTGTTGTGTGACTCTGGTAA	52	536	587	sense	2	1
**12**	E	AGTGCTTCAAGTAGTGTGTGCCCGTCTGTTGT	32	543	574	sense	2	21
**13**	D	AGTGCTTCAAGTAGTGTGTGCCCGTCTGTTGTGTGACTCTGGTAA	45	543	587	sense	2	1
**14**	A	TCAAGTAGTGTGTGCCCGTCTGTTGTGTGACTCTGGTAA	39	549	587	sense	2	14
**15**	H	GTAGTGTGTGCCCGTCTGTTGTGTGACTCTGGTAACTAGAGATCCCT	47	553	599	sense	2	2
**16**	D	GTGTGCCCGTCTGTTGTGTGACTCTGGTAACTAGAGAT	38	558	595	sense	2	4
**17**	C	GCCCGTCTGTTGTGTGACTCTGGTAACTAGAGAT	34	562	595	sense	2	2
**18**	H	CCCGTCTGTTGTGTGACTCTGGTAACTAGAGAT	33	563	595	sense	2	1
**19**	D	AAATCTCTAGCAGTGGCGCCCGAACAGGGACTT	33	623	655	sense	3	1
**20**	C	CCCTGTTCAGGCGCCA	16	651	636	antisense	3	1
**21**	F	AAAGGAGAGAGATGGGTGCGAGAGCGTCAGTATT	34	779	812	sense	4	2
**22**	F	AGAGAGATGGGTGCGAGAGCGTCAGT	26	784	809	sense	4	1
**23**	G	CTTTCCCCCTGGCCTTAGCCGAATTTTTTCCCATTTATCCAATTTT	46	867	822	antisense	5	9
**24**	J	CATATAGTATGGGCAAGCAGGGAGCTAGAACGTTTCGCAGTTAATCCTGGCCTGTTAGA	59	886	944	sense	6	1
**25**	J	CATATAGTATGGGCAAGCAGGGAGCTAGGACGTTTCGCAGTTAATCCTGGCCTGTTAGAATCATCAGAAGGCTGTAT	77	886	962	sense	6	1
**26**	J	CAAGCAGGGAGCTAGAACGTTTCGCAGATAATCCTGGCCTGTTAGA	46	899	944	sense	6	1
**27**	F	TTGTGTACATCAAAGGATAGAGGTAAAAGACACCAAGGAAGCTTT	45	1047	1091	sense	7	2
**28**	C	AAAGACACCAAGGAAGCTTTAGAAAAGATAGAGGAA	36	1072	1107	sense	7	2
**29**	J	GACCTGGCTGCTGTTTCCTGTGTCAGCTGCTGCTTGCTGTGCTTTTTTCTTACTTTTGGTTTG	63	1173	1111	antisense	8	5
**30**	H	GCTTCCTCATTGATGGTCTCTCTTAGCATTTGCATGGCTGCTTGATGT	48	1407	1368	antisense	9	2
**31**	H	ATTGATGGTCTCTTTTAGCATTTGCATGGCTGCTTGATGT	40	1415	1368	antisense	9	2
**32**	G	ACAGCTTCCTCGTTGATGGTCTCTTTTAGCATTTGCATGGCTGCTTGATGT	51	1418	1368	antisense	9	27
**33**	H	AACCAAGGGGAAGTGACATAGCAGGAACTACTAGTACCCTTCAG	44	1478	1521	sense	10	1
**34**	D	GTACCCTTCAGGAACAAATAGGATGGATGACAGATAATCCACCTATCCCAGTAGGAGAAATTTATAAAAGATGGATAATCCTG	83	1511	1595	sense	10	1
**35**	J	AACAAATAGGATGGATGACAAATAATCCACCTATCCCAGTAGGAGAAATCTATAAAAGATGGATAATCCTGGGGTTT	77	1523	1599	sense	10	1
**36**	J	AATAGTAAGGATGTATAGCCCTACCAGCATTCTGGACATAAGACAAGG	48	1605	1652	sense	11	2
**37**	H	AGCATTCTGGACATAAGACAAGGACCAAAGGAACCCTT	38	1630	1667	sense	11	1
**38**	F	GCATTCTGGACACAAGACAAGGACCAAAGGAACCCTTTAGAGACTATGTAGACCGGTTCTT	61	1631	1691	sense	11	1
**39**	G	CATTCTGGACGTAAGACAAGGACCAAAGGAACCCTTTAGAGACTATGTAGACCGGTTCTA	60	1632	1691	sense	11	1
**40**	F	ATTCTGGACATAAGACAAGGACCAAAGGAACCCTTTAGAGACTATGTAGACCGGTTCTA	59	1633	1691	sense	11	1
**41**	G	AGCCGAGCAAGCTTCACAGGAGGTA	25	1704	1728	sense	12	48
**42**	G	AACCTTGTTGGTCCAAAATGCGAACCCAGATCGTAAGA	38	1746	1783	sense	13	3
**43**	H	ACCTTGTTGGCCCAAAATGCGAACCCAGATTGTAAGATTA	40	1747	1786	sense	13	2

Seq No.	Library [(a)]	Sequence (5' - 3')	Length	Position HXB2 [(b)] Start	End	Polarity	Contig No.	sncRNA # of identical clones
**44**	C	ACCTTGTTGGTCCAAAATGCGAACCCAGATTGTAAGACTATTTTAAAAGCATTGGGACCAGCAGCCACACTAGA	74	1747	1820	sense	13	1
**45**	J	TACACTAGAAGAAATGATGACAGCATGTCAGGG	33	1812	1844	sense	13	6
**46**	H	GTCAGGGAGTGGGAGGACCCGGCCA	25	1838	1862	sense	13	3
**47**	J	AGGCAATTTTAGGAGCCAAAGAAAGATTGTTAAGTGTTTCAATTGTGG	48	1929	1976	sense	14	1
**48**	H	TTCAATTGTGGCAAAGAAGGGCACATAGCCAGAAATTGCAGGG	43	1966	2008	sense	14	4
**49**	H	TGCAGGGCCCCTAGGAAAAAGGGCTGTTGGAAATGTGGAAAGGAAGGA	48	2002	2049	sense	14	2
**50**	G	CACCAAATGAAAGATTGTACTGAGAGACAGG	31	2050	2080	sense	14	4
**51**	H	TCACTCTTTGGCAACGACCCCTCGTCACAATA	32	2260	2291	sense	15	1
**52**	F	GTATTAGAAGACATGGATTTGCCAGGAAGATGGAAA	36	2346	2381	sense	16	2
**53**	H	AACTCAAGACTTCTGGGAAGTTCAATTAGGAATACCACATCCCGCAGGGTT	51	2798	2848	sense	17	1
**54**	J	ACTTCTGGGAAGTTCAATTAGGAATACCACATCCCGCAGGGTTA	44	2806	2849	sense	17	1
**55**	J	AGAGAAAATCAGTAACAGTACTGGATGTGGGTGATGCATATTTTTCAGTT	50	2854	2903	sense	18	1
**56**	J	ATCAGTAACAGTACTGGATGTGGGTGATGCATATTTTTCAGTTCCCTTAGATAAAGACTTCAGGAAATA	69	2861	2929	sense	18	2
**57**	H	AACAGTACTGGATGTGGGTGATGCATATTTTTCAGTTCCCTTAGATA	47	2867	2913	sense	18	1
**58**	G	AACAGTACTGGATGTGGGTGATGCATATTTTTCAGTTCCCTTAGATAAAGACTTCAGGAAATA	63	2867	2929	sense	18	1
**59**	H	ACAGTACTGGATGTGGGTGATGCATATTTTTCAGTT	36	2868	2903	sense	18	1
**60**	H	ACAGTACTGGATGTGGGTGATGCATATTTTTCAGTTCCCTTAGATA	46	2868	2913	sense	18	1
**61**	J	ACAGTACTGGGTGTGGGTGATGCATATTTTTCAGTTCCCTTAGATAAAGACCTCAGGAAATA	62	2868	2929	sense	18	1
**62**	J	ATGTGCTTCCGCAGGGATGGAAAGGATCACCAGCAA	36	2989	3024	sense	19	1
**63**	C	ATCACCAGCAATATTCCAAAGTAGCATGACA	31	3014	3044	sense	19	1
**64**	J	AACATCTGTTGAGGTGGGGGTTTACCACACCAGACAAAAAACACCAG	47	3169	3215	sense	20	1
**65**	J	ACATCTGTTGAGGTGGGGATTTACCA	26	3170	3195	sense	20	1
**66**	J	AGTCAGATTTATGCAGGGATTAAAGTAAAGCAATTATGTAAACTCCTTAGGG	52	3351	3402	sense	21	1
**67**	H	TTATGTAAACTCCTTAGGGGAACCAAAGCACTAACAGAAG	40	3384	3423	sense	21	1
**68**	H	AGAAGTAATACCGCTAACAGAAGAAGCAGAGCTAGAACTGACAG	44	3419	3462	sense	21	1
**69**	A	AACATAGTGGACAGAATACTGGCAAGCCACCTGGATTCCTGAGTGGGAGTTTGTCAATA	59	3746	3804	sense	22	1
**70**	D	CCACCTGGATTCCTGAGTGGGAGTTTGTCAATA	33	3772	3804	sense	22	1
**71**	C	CCTGGATTCCTGAGTGGGAGTTTGTCAATA	30	3775	3804	sense	22	1
**72**	J	AATAGTAGCCAGCTGTGATAAATGTCAGCTAAAAGGAGAAGCCATGCATGGACAAGTAGTT	61	4334	4394	sense	23	2
**73**	J	CACATTTAGAAGGAAAAATTATCCTGGTAGCAGTTCATGTAGCCAGTGGATATATAGA	58	4426	4483	sense	24	1
**74**	F	CCATACAGACAATGGCAGCAATTTCACCAGTACTACGGTTAAGGCCGCCTGTTGGTGGGCTGGGATT	67	4568	4634	sense	25	1
**75**	J	CGGTTAAGGCCGCCTGTTGGTGGGCTGGGATCAAGCAGGAATTTGGCATTCCCTACA	57	4603	4659	sense	25	2
**76**	J	AACATCTTAAGACAGCAGTACAAATGGCAGTATTCATCCACAATTTTA	48	4738	4785	sense	26	1
**77**	H	CCGTTGCTATTATGTCTACTATTCTTTCCCCTGCACTGTA	40	4848	4809	antisense	27	1
**78**	F	GTATGTCCGTTGCTATTATGTCTACTATTCTTTCCCCTGCACTGTA	46	4854	4809	antisense	27	4
**79**	F	AAAGGACCAGCAAAGCTTCTCTGGAAAGGTGAAGGGG	37	4935	4971	sense	28	20
**80**	J	AGATAATAGTGATATAAAAGTAGTGCCAAGAAGAAAAGTAAAGATCATTAGGGATTA	57	4985	5041	sense	29	5
**81**	G	ACTATGAAAGCACTCATCCAAGAATAAGTTCAG	33	5168	5200	sense	30	32
**82**	F	ATAAGTTCAGAAGTACACATCCCACTAGGGGATGCTAGATTAGT	44	5191	5234	sense	30	1
**83**	F	AGGAGAAAGAGACTGGCATTTGGGTCAGGGAGTCTCCATAGAATGGAGGA	50	5262	5311	sense	31	1
**84**	H	AGACTGGCATTTGGGTCAGGGAG	23	5271	5293	sense	31	4
**85**	H	GACTGGCATTTGGGTCAGGGAGT	23	5272	5294	sense	31	9
**86**	F	TGACCTAGCAGACCAACTAATTCATCTGTATTACTTTGATTGTTTTTCAG	50	5340	5389	sense	32	2
**87**	G	TTCATCTGTATTACTTTGATTGTTTTTCAGCATCTGCTAT	40	5360	5399	sense	32	2
**88**	C	ATCTGTATTACTTTGATTGTTTTTCAGCATCTGCTATAAGAAATGCCATATTAGGACATATAGTTAGTCCTAGATGTGAA	80	5363	5442	sense	32	1
**89**	H	TTGTTTTTCAGAATCTGCTATAAGAAATGCCATATTAGGACATATAGTTAGTCCTAG	57	5379	5435	sense	32	1

Seq No.	Library [a]	Sequence (5' - 3')	Length	Position HXB2 [b] Start	End	Polarity	Contig No.	sncRNA # of identical clones
**90**	E	AAGAAATGCCATATTAGGACATACAGCTAGTCCTAGATGTGAATATCAAGCAGGACAT	58	5400	5457	sense	32	1
**91**	C	AAATGCCATATTAGGACATATAGTTAGTCCTAGATGTGAATATCAAGCAGGACATA	56	5403	5458	sense	32	1
**92**	D	AACTAACAGAGGATAGATGGAACAAGCCCCAGA	33	5543	5575	sense	33	1
**93**	C	ACACTAGAGCTTTTAGAGGAACTTAAAAGTGAAGCTGTTAGACATTTTCCTAGGGTCTGGCTTTT	65	5613	5677	sense	34	1
**94**	J	CATTTTCCTAGGGTCTGGCTCCATAGCTTAGGGCA	35	5655	5689	sense	34	13
**95**	H	CATTTTCCTAGGGTCTGGCTCCATAGCTTGGGGCAAT	37	5655	5691	sense	34	1
**96**	G	TACTGCAACAGCTGCTGTTTATTCATTTCAGAATTGGGTGTCAACAT	47	5746	5793	sense	35	16
**97**	H	AACAGCCGCTGTTTATTCATTTCAGAATTGGGTGTCAACAT	41	5752	5793	sense	35	1
**98**	E	GCTGCTGTTTATTCATTTCAGAATTGGGTGTCA	33	5756	5789	sense	35	1
**99**	G	AAATGGAGCCAGTAGATCCTAGCCTAG	27	5829	5855	sense	36	4
**100**	D	CTCCTATGGCAGGAAGAAGCGGAGACAGCGACGAAGAGCTCCTCAAGACA	50	5965	6014	sense	37	37
**101**	J	CTCTATCAAAGCAGTAAGTAGTGCATGTT	29	6033	6061	sense	38	1
**102**	J	CTCTATCAAAGCAGTAAGTAGTGCATGTTCA	31	6033	6063	sense	38	3
**103**	E	CAATAGTAGCATTAGTAGTAGCAATAATAATAGCAATAGTTGTGTGGTCCATAGTACTCATAGAATATAGGAAAATA	77	6084	6160	sense	39	3
**104**	H	AGTAGCATTAGTAGTAGCAATAATAATAGCAATAGTTGTGTGGT	44	6088	6131	sense	39	5
**105**	H	GTAGCATTAGTAGTAGCAATAATAATAGCAATAGTTGTGTGGTCCATA	48	6089	6136	sense	39	1
**106**	C	TAGTAGCAATAATAATAGCAATAGTTGTGTGGTCCATAGTACTCATAGAATATAGGAAAA	60	6099	6158	sense	39	1
**107**	H	AATAGCAATAGTTGTGTGGTCCATAGTACTCATAGA	36	6112	6147	sense	39	2
**108**	J	AAAATAGATAGGTTAATTGATAGAATAAGAGAGAGT	36	6173	6208	sense	40	1
**109**	H	ATTATCAGCACTTGTGGAGAGGGGGCACCTTGCTCCTTGGAAT	43	6241	6295	sense	41	1
**110**	H	AATCAGCACTTGTGGAGAGGGGGCACCTTGCTCCTTGGAA	40	6243	6294	sense	41	2
**111**	H	CTCCTTGGGATATTAATGATCTGTAGTGCTGTAGAA	36	6285	6320	sense	41	2
**112**	D	TGGAATAATAATGATCTGTAGTGCTGTAGAAAAGTTGTGGGTCACAGTCTAT	52	6290	6341	sense	41	1
**113**	C	ATATTAATGATCTGTAGTGCTGTAGAAAAGTTGTGGGTCACAGT	44	6294	6337	sense	41	1
**114**	J	ATATTAATGATCTGTAGTGCTGTAGAAAAGTTGTGGGTCACAGT	44	6294	6337	sense	41	2
**115**	H	AATAATGATCTGTAGTGCTGGAGAAAAGTTGTGGGTCACAGTCT	44	6296	6339	sense	41	5
**116**	D	TAATGATCTGTAGTGCTGTAGAAAAGTTGTGGGTCACAGTCT	42	6298	6339	sense	41	2
**117**	G	AATGATCTGTAGTGCTGTAGAAAAGTTGTGGG	32	6299	6330	sense	41	3
**118**	G	ATCTGTAGTGCTGTAGAAAAGTTGTGGGT	29	6303	6331	sense	41	12
**119**	J	CAGTCTATTGTGGGGTACCTGTGTGGAGAGAAGCAACCACCACTCTATTTTGTGCATCA	59	6334	6392	sense	41	1
**120**	C	GCATATGATACAGAGGTACATAATGTTTGGGCCACACATGCCTGTGTACCCACAGA	56	6402	6457	sense	42	8
**121**	H	TATGATACAGAGGTACATAATGTTTGGGCCACACATGCCTGTGTACCCACAGA	53	6405	6457	sense	42	1
**122**	E	ACATAATGTTTGGGCCACACATGCCTGTGTACCCACAGA	39	6419	6457	sense	42	2
**123**	E	ACATAATGTTTGGGCCACACATGCCTGTGTACCCACAGACCCTAACCCACAAGAAGTAGTATTGGAAAATGT	72	6419	6490	sense	42	6
**124**	H	AATGTTTGGGCCACACATGCCTGTATACCCACAGA	35	6423	6457	sense	42	1
**125**	D	ATGTTCGGGCCACACATGCCTGTGTACCCACAGA	34	6424	6457	sense	42	12
**126**	H	TTTGGGCCACACATGCCTGTGTACCCACAGA	31	6427	6457	sense	42	1
**127**	H	TTGGGCCACACATGCCTGTGTACCCACAGA	30	6428	6457	sense	42	2
**128**	D	CCACACATGCCTGTGTACCCACAGA	25	6433	6457	sense	42	2
**129**	E	CCACACATGCCTGTGTACCCACAGA	25	6433	6457	sense	42	1
**130**	A	ACACATGCCTGTGTACCCACAGA	23	6435	6457	sense	42	1
**131**	E	CACATGCCTGTGTACCCACAGA	22	6436	6457	sense	42	2
**132**	H	CACATGCCTGTGTACCCACAGA	22	6436	6457	sense	42	1
**133**	G	ACATGCCTGTGTACCCACAGA	21	6437	6457	sense	42	8
**134**	F	TAACATGTGGAAAAATAACATGGTAGAACAGATGCAGGAGGAT	43	6503	6545	sense	43	6
**135**	D	AACAGATACAGGAGGATATAATCAGTTTATGGGATCAAAGCCTAA	45	6529	6573	sense	43	1

Seq No.	Library [(a)]	Sequence (5' - 3')	Length	Position HXB2 [(b)] Start	End	Polarity	Contig No.	sncRNA # of identical clones
**136**	H	TAATCAGTTTATGGGATCAAAGCCTAAAGCCATGTGTAAAATTAA	45	6547	6591	sense	43	5
**137**	G	AATCAGTTTATGGGATCAAAGCCTAAAGCCATGTGTAAAATTTACCCCACTCTGTGTTACTTTA	64	6548	6613	sense	43	8
**138**	D	GTTTATGGGATCAAAGCCTAAAGCCATGTGTAAAATTAACCCCACTCTGTGTTA	54	6553	6606	sense	43	1
**139**	J	ACCCCACTCTGTGTTACTTTAAATTGCAAGGATGTG	36	6591	6626	sense	43	2
**140**	F	CTCTGTGTTACTTTAAATTGCAAGGATGTGAATGCTACTAATA	43	6597	6642	sense	43	14
**141**	J	CTTTAAATTGCAAGGATGTGAATGCTACT	29	6607	6635	sense	43	1
**142**	H	TTGCAATGATGTGAATGCTACTAATACCACTAGTGGTAGCGAGG	44	6614	6660	sense	43	1
**143**	J	GTGGTGATATTGAAAGAGCAGTTTTTATTTCT	33	6712	6680	antisense	44	4
**144**	J	AACTTGATGTAGTACCAATAGGATAATAATAATACCAGCTATAGGTTGATA	51	6757	6807	sense	45	1
**145**	A	CCCATACATTATTGTGCCCCGGCTGGTTTTGCGATTCTAAAGTGTAATG	49	6864	6912	sense	46	1
**146**	E	CATTATTGTGCCCCGGCTGGTTTTGCGATT	30	6870	6899	sense	46	5
**147**	D	CATTATTGTGCCCCGGCTGGTTTTACGATTCTAAAGTG	38	6870	6907	sense	46	4
**148**	C	CATTATTGTGCCCCGGCTGGTTTTGCGATTCTAAAGTGTAATG	43	6870	6912	sense	46	1
**149**	A	CATTATTGTGCCCCGGCTGGTTTTGCGATTCTAAAGTGTAATGATAAGACGTT	53	6870	6922	sense	46	4
**150**	A	CCCCGGCTGGTTTTGCGATTCTAAAGTGTAAT	32	6880	6911	sense	46	6
**151**	C	TTCTAAAGTGTAATGATAAGACCTTCAATGGAAAAGGACCATGTATTT	48	6898	6945	sense	46	1
**152**	J	TCTAAAGTGTAATGATAAGACGTTCAATGGAAAAGG	36	6899	6934	sense	46	3
**153**	C	ACAATGTACACATGGAATTAGGCCAGTAGTATCAACTCAACTGCTGCTAAATGGCAGTCTAGCAGA	66	6959	7024	sense	47	4
**154**	G	AATTAGATCTGACAATTTCACGAACAATGCTAAAACCA	38	7037	7074	sense	48	2
**155**	C	ATTAGATCTGACAATTTCACGAACAATGCTAA	32	7038	7069	sense	48	1
**156**	E	AATTGTAATGCACAGTTTTAATTGTGGAGGAGAATTTTTCTACTGT	46	7334	7379	sense	49	1
**157**	J	AACTGTTTAATAGTACTTGGAATAATAATACTGAAGGGTCAAATT	45	7390	7443	sense	50	1
**158**	B	ATAAACATGTGGCAGGAAGTAGGAAAAGCAATGTATG	37	7494	7530	sense	51	2
**159**	H	CATGTGGCAGGAAGTAGGAAAAGCAATGTATG	32	7499	7530	sense	51	3
**160**	D	ATGTGGCAGGAAGTAGGAAAAGCAATGTATG	31	7500	7530	sense	51	1
**161**	J	ATGTGGCAGGAAGTAGGAAAAGCGATGTATG	31	7500	7530	sense	51	1
**162**	D	GACCTGGAGGAGGAGATATGAGGGACAACTGGAGAAGTGA	40	7630	7669	sense	52	6
**163**	C	ACCTGGAGGAGGAGATATGAGGGACAATTGGAGAAGTG	38	7631	7668	sense	52	3
**164**	H	ACATCACTTCTCCAATTGCCCCTCATATCT	30	7672	7643	antisense	52	1
**165**	E	CTATTATTGTCTGGTATAGTGCAACAGCAGAACAATTTGCTGAGGGCTATTGAGGCGCAACAGCGTATGTTGCAACTCACAGTCTGGGG	89	7851	7939	sense	53	1
**166**	D	CTCTGGAAAACTCATTTGCACCACTGCTGTGCCTTG	36	8018	8053	sense	54	1
**167**	D	AACTCATTTGCACCACTGCTGTGCCTTGGAATGCTA	36	8026	8061	sense	54	5
**168**	H	CTGCTGTGCCTTGGAATGCTAGTTGGAGTAATAAATCTCTGGATA	45	8041	8085	sense	54	1
**169**	J	GATTTGGAATAACATGACCTGGATGGAGTGGGT	33	8087	8119	sense	55	1
**170**	E	AACATGACCTGGATGGAGTAGAAAAGAGAAATTGACAATTACACAAGCGAA	51	8097	8147	sense	55	1
**171**	J	TGACCTGGATGGAGTGGGAAAGAGAAATTTACAATTACACA	41	8101	8141	sense	55	4
**172**	J	AACAAGAATTATTGGAATTAGATAAATGGGCAAGTTTGTGGAATTGGTTTGACATA	56	8194	8249	sense	56	1
**173**	J	ATTGGAATTAGATAAATGGGCAAGTTTGTGGAATTGGTTTGACATA	46	8204	8249	sense	56	1
**174**	J	AGTTTGTGGAATTGGTTTGACATAACAAAATGGCTG	36	8226	8261	sense	56	1
**175**	E	AGGCTTGATAGGTTTAAGAATAGTTTTTACTGTACTTTCTATAGTGAATAGAGTTAGGCAGGGATTT	67	8294	8360	sense	57	3
**176**	C	TAGTTTTTACTGTACTTTCTATAGTGAATAGAGTTAGGCAGGGATACTCACCATTATCGTTTCAGA	66	8314	8379	sense	57	1
**177**	J	ATACTCACCATTATCGTTTCAGA	23	8357	8379	sense	57	2
**178**	H	CTCACCATTATCGTTTCAGA	20	8360	8379	sense	57	1
**179**	J	TCACCATTATCGTTTCAGA	19	8361	8379	sense	57	11
**180**	J	CCGTTCACTAATCGTTCGGATCTGTCTCTGTCTCTCTCT	39	8476	8438	antisense	58	3
**181**	E	TAATCGTCCGGATCTGTCTCTGTCTCTCT	29	8468	8440	antisense	58	1

Seq No.	Library [a]	Sequence (5' - 3')	Length	Position HXB2 [b] Start	End	Polarity	Contig No.	sncRNA # of identical clones
**182**	J	TTCACTAATCGTCCGGATCTGTCTCTGTCTCTCT	34	8473	8440	antisense	58	1
**183**	E	CGTTCACTAATCGTCCGGATCTGTCTCTGTCTCTCT	36	8475	8440	antisense	58	2
**184**	J	TTCACTAATCGTCCGGATCTGTCTCTGTCTCT	32	8473	8442	antisense	58	1
**185**	H	CAGATCCGGACGATTAGTGAACGGATT	27	8453	8479	sense	58	5
**186**	E	CACTTATCTGGGTCGACCTGCGGAGCCTGTG	31	8485	8515	sense	59	6
**187**	H	ACTTATCTGGGTCGACCTGCGGAGCCTGTG	30	8486	8515	sense	59	1
**188**	J	ACTTATCTGGGTCGACCTACGGAGCCTGTGCCTCTTCAGCTACCACCGCTTGAGAGACTTACTCTTGACTGTAACGAG	78	8486	8563	sense	59	1
**189**	C	CTCTTGACTGTAACGAGGATTGTGGAA	27	8547	8573	sense	59	4
**190**	H	GATTGTGGAACTTCTGGGACGCAGGGAGTGAGAAGTCCTGAAATATCGGTGGG	53	8564	8616	sense	59	1
**191**	G	CTTGCTCAATGCCACAGCCATA	22	8663	8684	sense	60	2
**192**	J	CTTGCTCAATGCCACAGCCATAG	23	8663	8685	sense	60	1
**193**	E	CAAAACGTAGTGTGCCTGGATGGTCTACTGTAAGGGA	37	8813	8849	sense	61	1
**194**	G	AAAACGTAGTGTGCCTGGATGGTCTACTGTAAGGGAAAG	39	8814	8852	sense	61	6
**195**	G	CAGGTCTCGAGATACTGCT	19	8907	8889	antisense	62	24
**196**	E	TTGCTCCGTGTTTTTCCAGGTCTCGAGATACTGCT	35	8923	8889	antisense	62	4
**197**	E	GATTGCTCCATGTTTTTCCAGGTCTCGAGATACTGCT	37	8925	8889	antisense	62	1
**198**	C	CGAGACCTGGAAAAACATGGAGCAATCACAAGTAGCAATACAGCAGCTACCAATGCTGATTGTGCCTGACTAGAAGCATA	80	8899	8978	sense	62	2
**199**	B	AAGCACAAGAGGATGAGGAGGTGGGTTTTCCAGTCAGACCTCAGGTA	47	8972	9018	sense	62	12
**200**	C	CCAATGACTTACAAGGGAGCTGTAGATCTTAGCCACTTTTTAAAAGA	47	9028	9074	sense	63	1
**201**	F	CACACAAGGCTACTTCCCTGATTGGCAGAACTACACACCAGGGCCAGGGGT	51	9144	9194	sense	64	1
**202**	G	TGATTGGCAGAACTACACACCAGGGCCAGGAATCAGATTTCCATTGACCTTTGGATGGTGCT	62	9162	9223	sense	64	16
**203**	E	CTACACACCAGGGCCAGGAATCAGATTTCCATTGACCTTTGGATGGTGCTTCAAGCTAG	59	9174	9232	sense	64	45
**204**	D	GATTTCCACTGACCTTTGGATGGTGCTTCAAGCTAGTACCAGTTGAGCCA	50	9197	9246	sense	64	1
**205**	J	CATTGACCTTTGGATGGTGCTTCAAGCTAGTACCAGTTGAGCCA	44	9203	9246	sense	64	3
**206**	D	CTGACCTTTGGATGGTGCTTCAAGCTAGTACCAGTTGAG	39	9205	9243	sense	64	2
**207**	D	CTGACCTTTGGATGGTGCTTCAAGCTAGTACCAGTTGAGCCA	42	9205	9246	sense	64	3
**208**	G	AACAAGCAGTTGTTCTCTCCTT	22	9290	9269	antisense	65	14
**209**	D	GCTTGTTACACCCTATGAGCCAGCATGGGATGGGCGA	37	9284	9320	sense	65	1
**210**	D	GCTTGTTACACCCTATGAGCCAGCATGGGATGGACGACCCGGAGAGGGAA	50	9284	9333	sense	65	7
**211**	E	ATGGGATAGAGGACCCGGAGAAGGAAGTGTTAGAGTGGAGGTTTGACAG	49	9308	9356	sense	65	11
**212**	H	TCATCACGTGGCCCGAGAGCTGCATCCGGAGTACT	35	9369	9403	sense	66	2
**213**	C	TCACGCGGCCCGAGAGCTGCATCCGGAGTACTACAAGGA	39	9372	9410	sense	66	1
**214**	D	TCACGTGGCCCGAGAGCTGCATCCGGAGTACTACAAGGA	39	9372	9410	sense	66	45
**215**	C	CGCCTCCCTGGAAAGT	16	9467	9452	antisense	67	1
**216**	H	CCTCCCGCGCAGGCCACG	18	9483	9466	antisense	67	1

[a] libraries A, C, E, G, and J are derived from macrophages and libraries B, D, F, and H from $CD4^+$ T-lymphocytes

[b] positions of sncRNAs are based on the reference HIV-1 strain HXB2 (GenBank accession number K03455)
