# Supplementary material for: Tailored enrichment strategy detects low abundant small noncoding RNAs in HIV-1 infected cells
Source: Retrovirology. 2012 Mar 29;9:27. doi: 10.1186/1742-4690-9-27 (PMC3341194; doi:10.1186/1742-4690-9-27)
Supplement: Additional file 4 — Table S4. Captured cellular miRNAs. [file 1742-4690-9-27-S4.PDF]

Table S4: Captured cellular miRNAs

Library [(a)]	miRNA	number of times a given miRNA was captured
A, K	hsa-let-7a	6
A, B, C, E, H, J, O, K	hsa-miR-21	36
A, E, H, J, O, K	hsa-miR-23a	31
J, K	hsa-miR-26a	3
A, K	hsa-miR-26b	2
A	hsa-miR-27a	1
A	hsa-miR-29a*	1
A, K	hsa-miR-34a	4
J	hsa-miR-92a	1
K	hsa-miR-93	1
A	hsa-miR-125a	1
K	hsa-miR-126	1
K	hsa-miR-146a	8
M	hsa-miR-146b	1
H	hsa-miR-155	2
E	hsa-miR-181a	1
J, A, O, K	hsa-miR-191	19
H	hsa-miR-210	1
M	hsa-miR-223*	1
A	hsa-miR-378a	3
A	hsa-miR-378d	1

[(a)] Libraries A, C, E, J, M and O are derived from macrophages and libraries B and H from $CD4^+$ T-lymphocytes.
